# Differential functional genomic effects of anti-inflammatory phytocompounds on immune signaling

**DOI:** 10.1186/1471-2164-11-513

**Published:** 2010-09-24

**Authors:** Shao-Chih Chiu, Shan-Wen Tsao, Pei-Ing Hwang, Staniforth Vanisree, Yi-An Chen, Ning-Sun Yang

**Affiliations:** 1 Graduate Institute of Immunology, China Medical University, 91 Hsueh-Shih Rd., Taichung 40402, Taiwan; 2 Center for Neuropsychiatry, China Medical University Hospital, 2 Yude Rd., Taichung 40447, Taiwan; 3 Agricultural Biotechnology Research Center, Academia Sinica, 128 Sec. 2, Academia Rd., Nankang, Taipei 11529, Taiwan; 4 Department of Life Science, National Central University, 300 Jhongda Rd., Jhongli City, Taoyuan County 32001, Taiwan; 5 Institute of Statistical Science, Academia Sinica, 128 Sec. 2, Academia Rd., Nankang, Taipei 11529, Taiwan

## Abstract

**Background:**

Functional comparative genomic analysis of the cellular immunological effects of different anti-inflammatory phytocompounds is considered as a helpful approach to distinguish the complex and specific bioactivities of candidate phytomedicines. Using LPS-stimulated THP-1 monocytes, we characterize here the immunomodulatory activities of three single phytocompounds (emodin, shikonin, and cytopiloyne) and a defined phytocompound mixture extracted from *Echinacea *plant (BF/S+L/Ep) by focused DNA microarray analysis of selected immune-related genes.

**Results:**

Shikonin and emodin significantly inhibited the early expression (within 0.5 h) of approximately 50 genes, notably cytokines TNF-α, IL-1β and IL-4, chemokines CCL4 and CCL8, and inflammatory modulators NFATC3 and PTGS2. In contrast, neither cytopiloyne nor BF/S+L/Ep inhibited the early expression of these 50 genes, but rather inhibited most late-stage expression (~12 h) of another immune gene subset. TRANSPATH database key node analysis identified the extracellular signal-regulated kinase (ERK) 1/2 activation pathway as the putative target of BF/S+L/Ep and cytopiloyne. Western blot confirmed that delayed inactivation of the ERK pathway was indeed demonstrable for these two preparations during the mid-stage (1 to 4 h) of LPS stimulation. We further identified ubiquitin pathway regulators, E6-AP and Rad23A, as possible key regulators for emodin and shikonin, respectively.

**Conclusion:**

The current focused DNA microarray approach rapidly identified important subgenomic differences in the pattern of immune cell-related gene expression in response to specific anti-inflammatory phytocompounds. These molecular targets and deduced networks may be employed as a guide for classifying, monitoring and manipulating the molecular and immunological specificities of different anti-inflammatory phytocompounds in key immune cell systems and for potential pharmacological application.

## Background

Inflammation is the result of a cascade of physiological and immunological reactions that aim to localize toxic materials, fight pathogens and prevent tissue injury [1]. The inflammatory response consists of the sequential release of mediators including inflammatory cytokines and the recruitment of circulating leukocytes that become activated at the inflammatory site and release further mediators. In most cases, macrophage activation constitutes the key orchestration and regulation event of the inflammatory response [2].

Analysis of the kinetics of cytokine production during the inflammatory response reveals that macrophage activation is the product of an underlying process that impacts the genome within minutes and continues for several hours. The transcriptional regulation of gene expression is the mechanistic foundation of macrophage activation [3-5]. At the onset (~0.5 h) and in the early stages (within the first 2 h) of an inflammatory response, NF-κB, signal transducer and activator of transcription (STAT), activator protein-1 (AP-1), and CCAAT enhancer-binding protein (CEBP) control macrophage gene expression [5,6]. A secondary response or mid-term stage commences around 4 h, which primes the immune system for the resolution, and there is a final late stage response around 12 h after stimulus [7]. The interplay of these three stages thus determines the outcome of the specific and/or the overall inflammatory responses [5,8]. Detailed and mechanistic information concerning the integration of the systems involved in these events is useful not only for studies of immune-cell signaling mechanisms but also for the development of remedies (e.g., phytomedicines) to control excessive inflammation. We hypothesized at the outset of this study that different phytochemicals with reputed anti-inflammatory activities may exhibit distinctive patterns of effects and kinetics as they intervene in specific steps in the inflammatory cascade, and that such phytochemicals may thus be subgrouped on those grounds, at the pharmacogenomic level, for systematic mechanism studies or therapeutic applications.

The apparently integrated and programmed patterns of gene expression regulating the various steps of an inflammatory response make them a desirable target system for studying functional genomics of innate immunity. Currently, little comparative studies on the anti-inflammatory activities of phytocompounds/herbal extracts are available. Many phytocompounds are believed to be immunomodulatory, we and others have recently demonstrated such activities for a series of anti-inflammatory phytocompounds including shikonin, an inhibitor of TNF-α mRNA maturation [9] or transcription [6], and emodin, which represses the inflammatory response [10,11]. Another unique immuno-modulatory compound, cytopiloyne, recently isolated from the Asteraceae plant, *Bidens pilosa *[12], has also been reported to decrease the symptoms of autoimmune disease in mouse type I diabetes [13]. We have observed that both emodin and cytopiloyne can effectively modulate human dendritic cell function (unpublished results). In addition to these pure phytocompounds, we also reported earlier that a stem and leaf extract of *Echinacea purpurea *is anti-inflammatory in dendritic cells [14], which suggests that some complex herbal preparations may affect a spectrum of immune cell types during inflammation.

An appropriate model system to study macrophage activation is to investigate the response to lipopolysaccharide (LPS) challenge in THP-1 cells, an immortalized human monocyte/macrophage cell line that closely resembles PBMC-derived macrophages [3,15-17]. LPS, a molecular correlate of bacterial infection, binds directly to Toll-like receptor 4 to trigger multiple signaling cascades including those mediated through NF-κB and the Janus N-terminal kinase (JNK) and p38 kinase pathways [18]. LPS elicits the expression of multiple macrophage pro- and anti-inflammatory cytokines, and the resulting effects may be protective or deleterious. Therefore, the LPS-induced THP-1 cells provide a good inflammatory model system that can reflect macrophage activation induced by gram (-) bacteria and/or the related acute-inflammation responses and sepsis [3]. The activation of particular genes in these inflammatory response pathways is particularly amenable to study by functional genomic approaches such as focused DNA microarray, which uses an array of a limited subset of genes [3,19]. Here, we demonstrated the utility of this approach in characterization of the effects of different types of phytocompounds on monocyte gene expression patterns. Our findings also led us to hypothesize a number of master switch molecules in these immune cells that can respond differentially, at the signaling network level, to distinct groups of candidate phytomedicines.

## Results

### Determination of test phytocompound cytotoxicity in THP-1 cells

**Figure 1:**
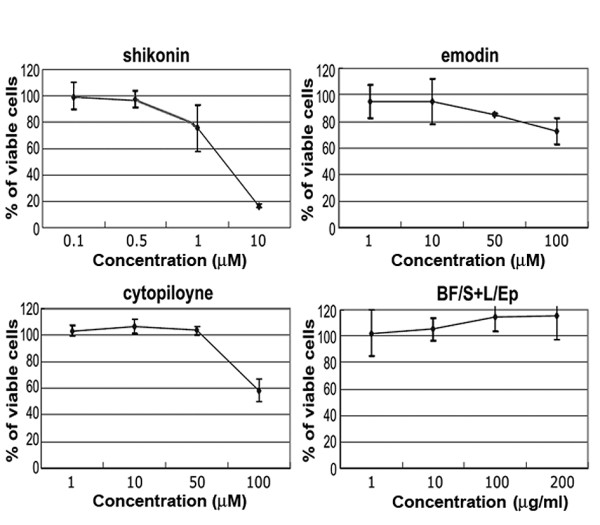
**Determining the non-toxic concentrations of test phytocompounds in THP-1 cells**. The highest non-toxic concentrations of test phytocompounds (shikonin, emodin, cytopiloyne and BF/S+L/Ep extract) were determined by MTT assay. THP-1 cells were cultured with different phytocompounds for 48 h. Assays were carried out in triplicate.

The immune modulatory effects of known anti-inflammatory phytocompounds and extracts were examined in the human monocytic cell line THP-1. The cytotoxicity of the test phytocompounds (shikonin, emodin, cytopiloyne and BF/S+L/Ep) was determined by MTT assay following culture with various concentrations of the compounds for 48 h (Figure 1). The highest concentrations that led to no significant decrease in cell viability (shikonin 0.5 μM, emodin 10 μM, cytopiloyne 20 μM, and BF/S+L/Ep 100 μg/ml) were used in subsequent experiments. Three phytochemicals (shikonin, emodin and cytopiloyne) were isolated, obtained, and tested as single, structurally known chemical compounds. Each of these compounds has been previously shown to modulate certain immunological bioactivities [9-13,20]. Shikonin is the active compound identified from a traditional medicinal herb, *Lithospermum erythrorhizon*. Emodin is an active compound presents in *Rheum officinale*. Cytopiloyne is an active compound isolated from the plant *Bidens pilosa*. BF/S+L/Ep was named as the butanol partitioned fraction (BF) of the stem + leaf (S+L) tissue extracts of the *E. purpurea *(Ep) plant. We have previously shown that this fraction may confer an immune-modulatory effect in human dendritic cells [14,20].

### Effect of phytocompounds on LPS-induced gene expression

To determine the effects of test phytocompounds/extracts on the LPS-induced inflammatory response in THP-1 cells, we compared the gene expression profiles of cells treated with LPS only and cells co-treated with LPS and test phytocompounds at different time points. Total RNA was collected at the indicated time points for focused microarray analysis as described previously [14].

**Figure 2:**
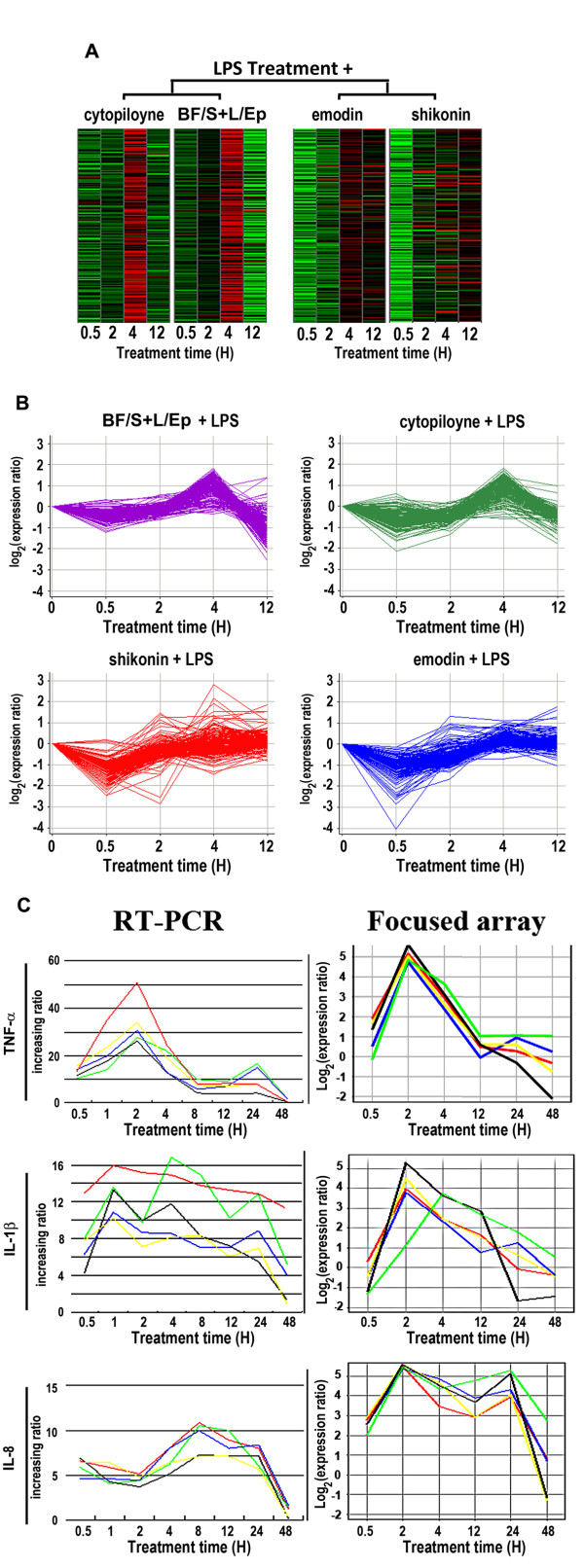
**Focused array analysis**. The effect of 4 immunomodulatory herbal derivatives on LPS-induced THP-1 cells. **(A) **Heat map representation of the expression profiles of 191 immune-related genes in THP-1 cells after the indicated treatments. Note the overall similarity between *Echinacea *BF/S+L/Ep and cytopiloyne treatments. **(B) **Time profile of test compound effects on LPS-stimulated gene expression compared to LPS stimulation only. **(C) **Correlation between gene expression patterns obtained from RT-PCR (left column) and focused array (right column). Kinetics of the expression of TNF-α, IL-1β, and IL-8 genes were analyzed by RT-PCR and microarray. Red: LPS only; green: shikonin + LPS; black: emodin + LPS; blue: cytopiloyne + LPS; yellow: BF/S+L/Ep + LPS. The RT-PCR ratio was relative to the gene expression in vehicle alone at the same time point. The Y-axis in the focused array is shown in log_2 _scale.

In LPS-stimulated THP-1 cells, 35 genes were either up- or down-regulated (data not shown) more than threefold compared to untreated cells (i.e., expression of these genes increased to more than 300% of normal expression, or decreased to less than 33% of normal expression levels). Two anti-inflammatory compounds, shikonin and emodin, inhibited the early LPS-induced threefold increase of pro-inflammatory gene expression, but cytopiloyne and BF/S+L/Ep did not show similar inhibitory effects (Figure 2B) at the early stage of inflammatory response. Shikonin significantly inhibited the expression of several genes (Table 1), including genes for cytokines (TNF-α, IL-1β and IL-4), chemotaxis and cell migration genes (CCL4 and CCL8) and inflammatory response genes (NFATC3 and PTGS2). Emodin significantly inhibited the increase in expression of several common genes, including cytokines (IL-1β and IL-4), and inflammatory response genes (NFATC3 and PTGS2) within 0.5 h of exposure (Table 1).

**Table 1:** Genes down-regulated ≥3-fold by shikonin or emodin at 0.5 hours.

			Gene Expression Ratio (Test + LPS): LPS only			
	Gene Symbol	Gene ID	shikonin	emodin	cytopiloyne	BF/S+L/Ep
**Cytokines**	IL1B	3553	0.32	0.35	0.59	0.59
	
	IL4	3565	0.25	0.2	0.92	0.81
	
	IL13	3596	0.36	0.33	0.6	0.71
	
	IRF1	3659	0.23	0.36	1.52	0.7
	
	KITLG	4254	0.31	0.34	1.03	0.74
	
	TNF	7124	0.24	0.68	0.38	0.85
	
	TNFSF9	8744	0.34	0.32	0.62	0.8

**Chemotaxis & Cell Migration**	CDH12	1010	0.22	0.34	0.23	0.45
	
	CCR2	1231	0.24	0.17	0.82	0.88
	
	CCR3	1232	0.26	0.38	0.49	0.55
	
	CCR4	1233	0.29	0.35	1.01	0.86
	
	CCBP2	1238	0.28	0.48	0.44	0.56
	
	ICAM2	3384	0.29	0.28	0.73	0.79
	
	ITGAE	3682	0.34	0.32	0.55	0.59
	
	CCL4	6351	0.3	0.64	0.49	0.87
	
	CCL8	6355	0.33	0.35	1.03	0.81
	
	CCL22	6367	0.27	0.28	0.72	0.64
	
	SELL	6402	0.26	0.91	0.58	1.15
	
	SELP	6403	0.24	0.18	0.44	0.57
	
	CXCR4	7852	0.39	0.19	0.59	1.26
	
	CD207	50489	0.26	0.41	0.6	0.67
	
	CLEC4A	50856	0.49	0.33	0.68	0.89
	
	CLEC1A	51267	0.3	0.47	0.32	0.54

**Inflammatory Responses**	CD1D	912	0.36	0.18	0.52	0.91
	
	CD80	941	0.31	0.39	0.33	0.52
	
	CSF3R	1441	0.49	0.33	0.69	0.82
	
	IL2RA	3559	0.25	0.23	0.95	0.86
	
	IL8RB	3579	0.27	0.34	0.64	0.53
	
	LIFR	3977	0.27	0.48	0.32	0.6
	
	MAP3K3	4215	0.3	0.27	0.78	0.73
	
	NFATC3	4775	0.18	0.06	0.8	1.15
	
	MAPK7	5598	0.28	0.26	1.09	0.66
	
	PTGS1	5742	0.36	0.3	0.72	0.82
	
	PTGS2	5743	0.25	0.17	1.03	0.93
	
	SLAMF1	6504	0.33	0.58	0.37	0.67
	
	STAT1	6772	0.29	0.2	1.04	0.8
	
	LILRB4	11006	0.18	0.16	0.94	0.78

**Other**	BCL2	596	0.32	0.4	0.7	0.65
	
	BCL2L1	598	0.31	0.31	0.98	0.61
	
	ETS1	2113	0.35	0.17	0.91	0.84
	
	FUT7	2529	0.35	0.32	0.78	0.57
	
	NR3C2	4306	0.33	0.48	0.89	0.66
	
	NGFR	4804	0.32	0.34	0.73	0.68
	
	TAP2	6891	0.2	0.14	0.72	0.85
	
	TFRC	7037	0.29	0.37	1.01	0.72

At 4 h after exposure, both cytopiloyne and BF/S+L/Ep treatments up-regulated the expression of cytokines (CSF1, IL5 and TNFSF14) and cell-migration related genes (including ITGAX and MADCAM1) (Table 2). At the late stage of the LPS-induced inflammatory response (12 h after exposure), BF/S+L/Ep significantly inhibited (more than threefold decrease in expression ratio) several inflammation response genes (GATA3, NFATC3, PTGS2), cytokines (including IL-4), and chemotaxis and cell adhesion genes (such as CCR2, CCR3, CXCR4 and ITGA2) (Table 3).

**Table 2:** Genes up-regulated ≥3-fold by BF/S+L/Ep or cyotpiloyne at 4 hours.

			Gene Expresion Ratio (Test + LPS): LPS only			
	Gene Symbol	Gene ID	shikonin	emodin	cytopiloyne	BF/S+L/Ep
**Cytokines**	CSF1	1435	0.91	1.69	3.54	3.12
	
	IL5	3567	0.87	1.35	2.77	3.01
	
	TNFSF14	8740	1.01	1.36	2.67	3.03

**Chemotaxis & Cell Migration**	CCR1	1230	0.87	1.3	2.91	3.09
	
	CCR8	1237	0.78	1.48	2.76	3.14
	
	ITGAX	3687	1.08	1.38	2.88	3.19
	
	CXCR4	7852	1.21	1.63	3.33	2.63
	
	MADCAM1	8174	0.82	1.45	3.04	3.39

**Inflammatory Responses**	CD14	929	0.9	1.56	3.24	3.26
	
	MAP2K4	6416	1.25	1.4	2.59	3.56
	
	TLR3	7098	0.91	1.33	2.35	3.06
	
	IL1R2	7850	0.91	1.49	2.64	3.13
	
	TNFRSF10D	8793	0.84	1.4	2.86	3.28
	
	TBX21	30009	0.66	1.34	2.89	3.2

**Other**	SHC1	6464	0.89	1.22	2.4	3.2
	
	ADAMDEC1	27299	1.14	1.79	2.71	3

**Table 3:** Genes down regulated ≥3-fold by BF/S+L/Ep or cyotpiloyne at 12 hours.

			Gene Expresion Ratio (Test + LPS): LPS only			
	Gene Symbol	Gene ID	shikonin	emodin	cytopiloyne	BF/S+L/Ep
**Cytokines**	IL4	3565	1.12	0.94	0.68	0.25
	
	IRF1	3659	2.01	0.87	0.78	0.27
	
	TNFRSF11B	4982	0.93	0.81	0.66	0.26
	
	TNFRSF10C	8794	1.04	0.96	0.86	0.32

**Chemotaxis & Cell Migration**	CDH1	999	1.07	0.81	0.74	0.27
	
	CDH12	1010	0.6	0.49	0.53	0.17
	
	CCR2	1231	1.24	1.17	0.45	0.28
	
	CCR3	1232	1.06	0.68	0.57	0.25
	
	CCBP2	1238	0.98	0.83	0.69	0.22
	
	ITGA2	3673	0.96	0.77	0.69	0.28
	
	ITGB1	3688	0.9	1.69	0.72	0.32
	
	CCL2	6347	0.7	0.7	0.29	0.7
	
	CXCR4	7852	1.04	1.69	0.6	0.32

**Inflammatory Responses**	CD80	941	1.71	1.09	0.55	0.3
	
	GATA3	2625	1.15	0.88	0.72	0.26
	
	IL7R	3575	1.67	0.88	0.73	0.3
	
	IL8RB	3579	0.83	0.64	0.62	0.26
	
	ITK	3702	1.07	0.95	0.79	0.3
	
	LIFR	3977	1	0.84	0.74	0.27
	
	NFATC3	4775	2.07	3.11	0.67	0.32
	
	MAP2K5	5607	1.31	1.03	0.65	0.33
	
	PTGS2	5743	1.26	0.97	0.63	0.29
	
	SLAMF1	6504	1.04	0.74	0.59	0.3
	
	IGSF6	10261	0.58	0.87	0.33	0.67
	
	NFATC2IP	84901	1.1	0.78	0.66	0.22

**Other**	ANXA3	306	0.97	0.88	0.65	0.33
	
	BCL2	596	1.05	1.19	0.87	0.29
	
	MPO	4353	0.84	0.77	0.68	0.27
	
	C1orf61	10485	1.19	1.02	0.7	0.31
	
	CLEC1A	51267	0.8	0.69	0.6	0.25

### Comparison of gene expression patterns among four different treatments

For gene clustering analyses, we first applied the hierarchical clustering method using the UPGMA (unweighted average) program. The gene expression patterns, as shown in the heat map in Figure 2A were then arranged to compare the similarities and differences between the experimental groups. While shikonin and emodin displayed a randomized pattern in heat map representations of the gene expression profiles in the focused array, BF/S+L/Ep treatment and cytopiloyne treatment shared a strikingly similar pattern (Figure 2A, 2B). We thus used RT-PCR analysis of three important inflammatory response signature genes, TNF-α, IL-8 and IL-1β, to confirm the data obtained from the microarray analyses, and found gene expression patterns similar to those observed in focused arrays (Figure 2C). Taken together, these results (Figure 2B, 2C) lead us to suggest that the data from our microarray assays represented meaningful gene expression patterns that can be verified by independent gene expression assay systems.

**Figure 3:**
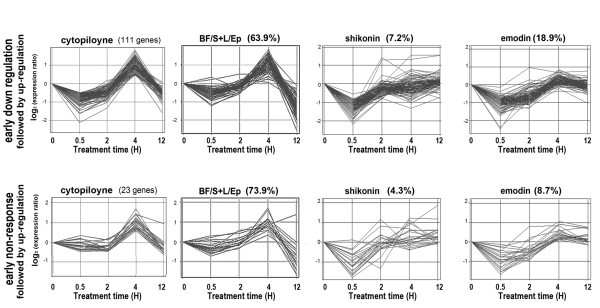
**Gene regulation patterns of phytocompounds clustered by different kinetics of expression ratio (compared to LPS stimulation alone)**. The number of genes in each mode affected by cytopiloyne is shown above each left panel, while the percentage of these genes with an expression regulation mode similar to the other three test compounds is shown above. **(A) **The cluster of "early down-regulation followed by up-regulation". **(B) **The cluster of "early non-response followed by up-regulation". The Y-axis, in log_2 _scale, shows the ratio of gene expression level between "LPS with compound treatment" and "LPS stimulation alone".

Next, we clustered the genes into regulation modes according to the four different patterns of changes in their expression ratios observed after cytopiloyne treatment following LPS stimulation. As stated previously, a predominant trend of up-regulation was observed at the 4 h time point. Nevertheless, the early down-regulation response of many of the genes allowed us to cluster the majority of the genes into 3 distinct groups of regulation mode, namely "early down-regulation followed by up-regulation" (Figure 3A), "early non-response followed by up-regulation" (Figure 3B), and "delayed down-regulation followed by up-regulation" (data not shown). Individual genes that did not fit into any of these three modes were grouped into a fourth classification, "other" (data not shown). We then compared the gene expression patterns seen after cytopiloyne treatment with the gene expression patterns seen after the other three treatments and calculated the degree of similarity as the percentage of genes that fell into the same regulation mode as cytopiloyne (Figure 3A, 3B). With BF/S+L/Ep treatment, the majority of the genes in the "early non-response" group (73.9%) and "early down-regulation" group (63.9%) fell into the same regulation mode as cytopiloyne. With shikonin and emodin, the fractions of genes with regulation modes corresponding to cytopiloyne were much lower: "early non-response" group, 4.3% and 8.7%, respectively; and "early down-regulation" group, 7.2% and 18.9%, respectively (Figure 3A, 3B). In the "delayed down" group, only 3.4%, 3.4%, and 24.1% of the genes affected in the BF/S+L/Ep, shikonin, and emodin treatments, respectively, displayed the same regulation mode as cytopiloyne (data not shown).

**Figure 4:**
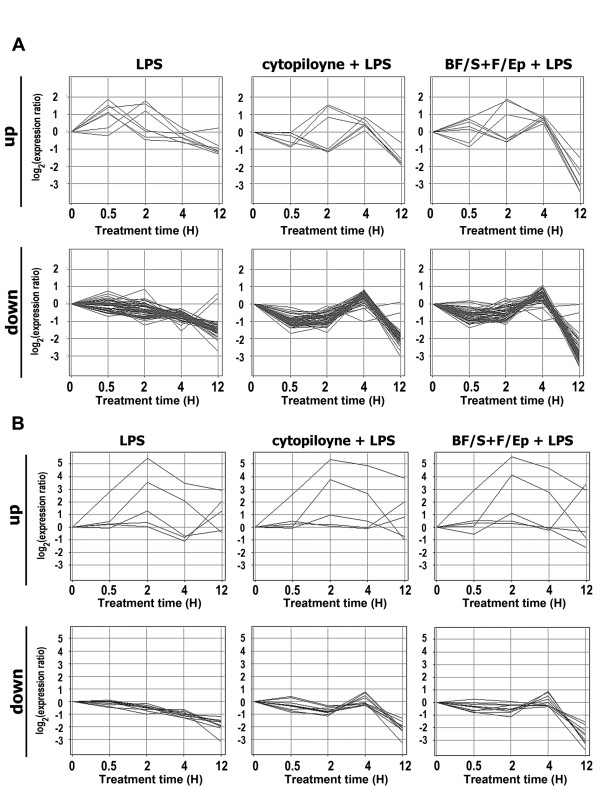
**Sub-grouping the expression profile of the genes regulated in a similar mode by BF/S+L/Ep and cytopiloyne treatments**. Two patterns of LPS-induced kinetic gene expression were characterized into "Up" (first panel) and "Down" (second panel). The second and third columns represent cytopiloyne or BF/S+L/Ep treatment, respectively. **(A) **Expression profiles of the genes corresponding to the "early down-regulation" group in cytopiloyne treatment. **(B) **Expression profiles of the genes corresponding to the "early non-response" group.

We were curious about the detailed mechanism responsible for the similarity in the effects of BF/S+L/Ep and cytopiloyne. For this purpose, we compared the expression profiles of the genes sharing common regulation modes in both BF/S+L/Ep and cytopiloyne treatment. Genes that displayed "early down-regulation" modes were subsequently classified into 2 types of expression profiles (Figure 4A, "up" and "down") according to the initial response of the gene in comparison with LPS treatment in THP-1 cells alone. Strikingly, all genes in the up-regulation sub-group showed sustained up-regulation after both cytopiloyne and BF/S+L/Ep treatments (Figure 4A, top panel). Genes that were down-regulated by LPS-only treatment produced the typical up-regulation pattern at 4 h with the Asteraceae preparations (cytopiloyne and BF/S+L/Ep) (Figure 4A, lower panel). The same scenario was observed in analysis of those genes displaying "early non-response" mode (Figure 4B, lower panel).

### Signaling molecules and associated pathways that may be involved in the modulation of LPS-induced inflammatory response

**Figure 5:**
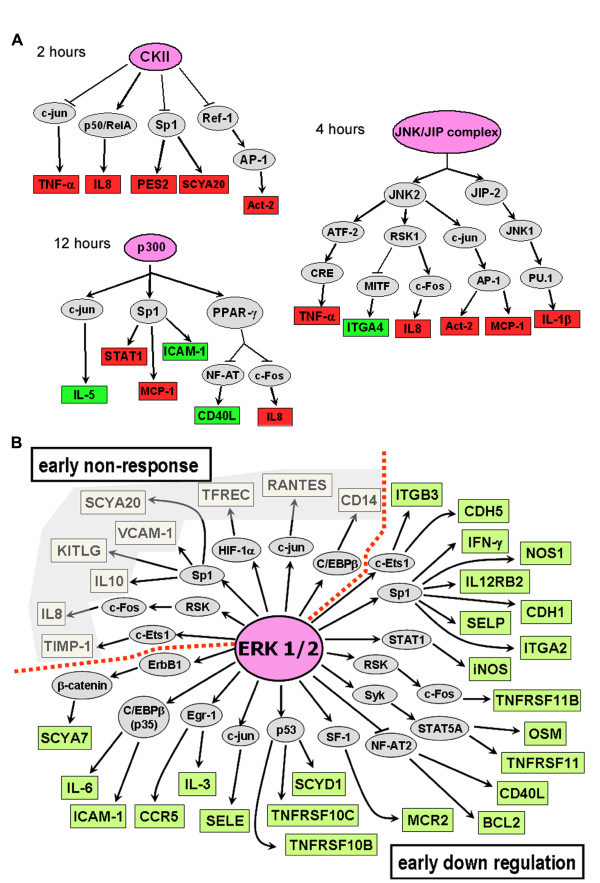
**Candidate master switch molecules and signaling pathways involved in LPS-induced inflammatory response of monocyte THP-1 cells**. **(A) **Key node analysis was used to assess the possible signaling pathways or potential interactions in response to LPS stimulation only, at different time points. Pink circle: the putative master regulator/molecule; red box: the up-regulation (≥ 3-fold) of gene expression induced by LPS compared to vehicle treatment; green box: the down-regulation (≥ 3-fold) of gene expression induced by LPS compared to the vehicle treatment; gray circle: assigned molecule in the putative pathway; **↑**: activation; **⊥**: inhibition. **(B) **TRANSPATH analysis of common signaling denominators. Genes with the same expression mode in the "early non-response" and "early down-regulation" groups in both BF/S+L/Ep and cytopiloyne treatments were analyzed. Only 4 hierarchical levels of regulation were accepted.

To find out the possible signaling pathways involved in the different gene expression patterns observed in cytopiloyne, BF/S+L/Ep, shikonin and emodin treatments, we analyzed the microarray data using the TRANSPATH database [21]. First, we analyzed those genes whose expression was up- or down-regulated more than threefold after 2, 4, and 12 h of LPS-only treatment to verify the processing steps in the TRANSPATH database. Three key molecules and signaling pathways were observed: CKII (casein kinase II), JNK/JIP and p300 were the target molecules at 2, 4 and 12 h time points respectively (Figure 5A). In this light, we reasoned that a possible target for Asteraceae preparations could be a common signaling molecule upstream of the genes sharing same expression modes. We then subjected selected genes from two groups (Figure 3A,3B) to key node analysis, which identified the ERK1/2 pathway as a single common denominator at no more than 4 steps of hierarchical gene regulation at 4 h (Figure 5B).

**Figure 6:**
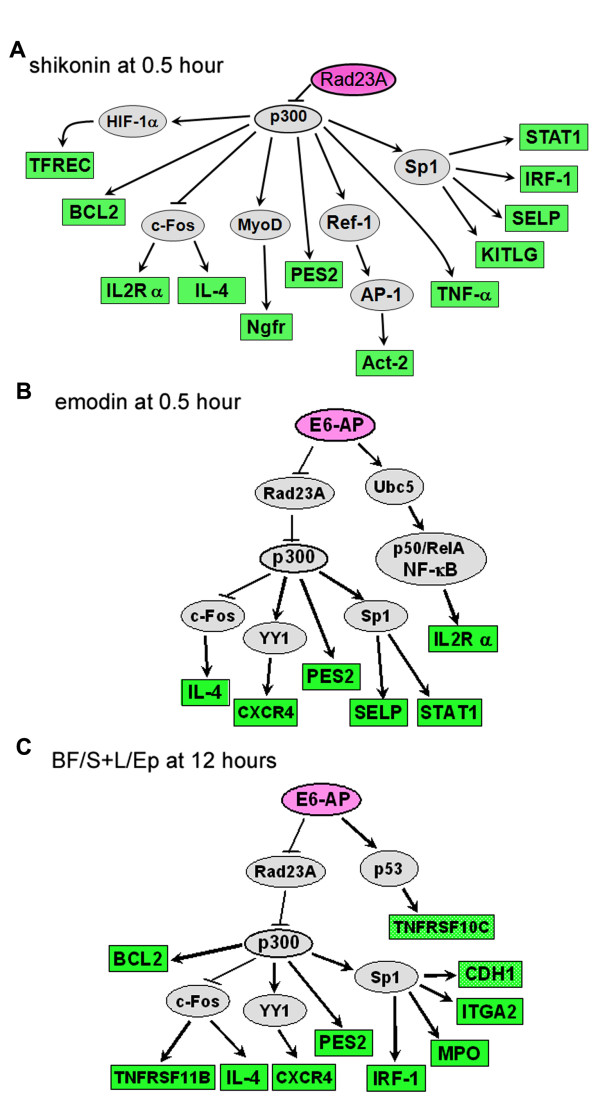
**Identification of putative signaling regulators for immune-modification**. Key node analysis in the TRANSPATH database for putative unique signaling master regulator/molecule at 0.5 h with significant inhibition of expression (≥3-fold decrease) in LPS-induced THP-1 cells co-treated with **(A) **shikonin, **(B) **emodin, or **(C) **BF/S+L/Ep at 12 h. Less than four hierarchical levels of regulation were accepted and selected. Pink circle: the putative master regulator/molecule; green box: the ratio of gene expression seen with the phytocompounds plus LPS compared to LPS only; gray circle: assigned molecule in the putative pathway; **↑**: activation; **⊥**: inhibition.

The shikonin- and emodin-affected genes were analyzed by the same method, and a specific molecule and signaling pathway was observed for each treatment. The possible master regulator in the treatment with shikonin plus LPS at 0.5 h was identified by a signaling database search as Rad23A (Figure 6A). The possible master regulator in the emodin plus LPS treatment at 0.5 h was the ubiquitin protein ligase E3A (E6-AP) (Figure 6B). For treatment with cytopiloyne or BF/S+L/Ep, there was little or no significant change in gene expression at the early stage. However, among all four phytocompounds tested, only the BF/S+L/Ep treatment showed a significant inhibition of LPS-stimulated gene expression increase in our focused array at 12 h (Table 3). Key node analysis of genes with significant down-regulation pointed to E6-AP as a possible master regulator (Figure 6C).

**Figure 7:**
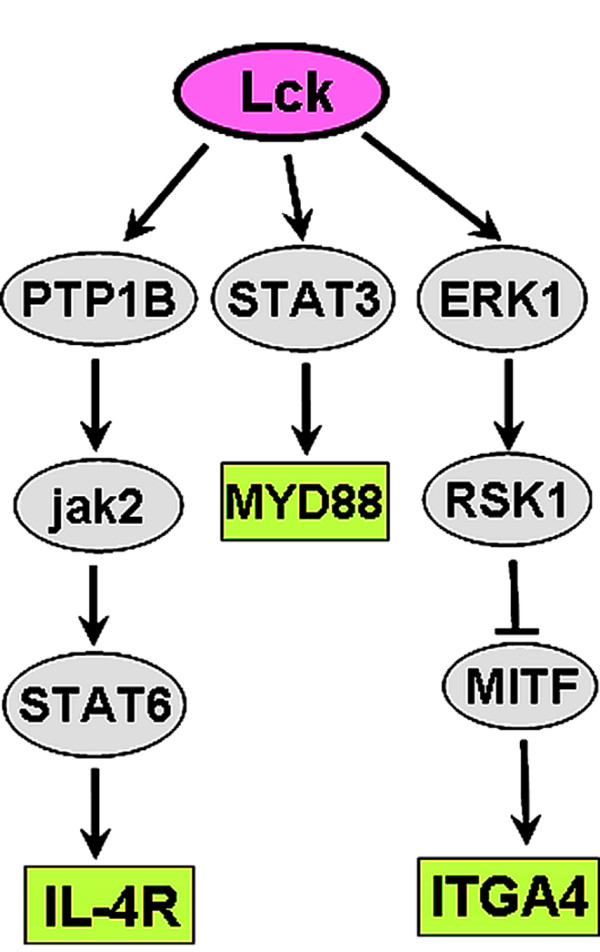
**Identification of putative common signaling denominators shared by treatments with BF/S+L/Ep and cytopiloyne**. Key node analysis in TRANSPATH database for putative unique signaling denominator of the "delayed down-regulated" group between BF/S+L/Ep and cytopiloyne. Only four hierarchical levels of regulation were accepted.

Having identified ERK1/2 as a putative target of both BF/S+L/Ep and cytopiloyne (Figure 5B), we next aimed to distinguish the mechanistic difference between these two preparations. "Delayed down-regulation" was the only group of genes with almost no intersection in expression mode between BF/S+L/Ep and cytopiloyne (data not shown). The genes in this group were again analyzed using the TRANSPATH database, in search of upstream effector molecules that were not present in the other two expression modes (Figure 3A, 3B). This analysis identified one potential pathway pointing to a key regulator, Lck, a member of the Src family of protein tyrosine kinases important in T-lymphocyte activation and differentiation (Figure 7).

### Delay in inactivation of the ERK pathway during the mid-stage (~4 hours) of LPS stimulation

**Figure 8:**
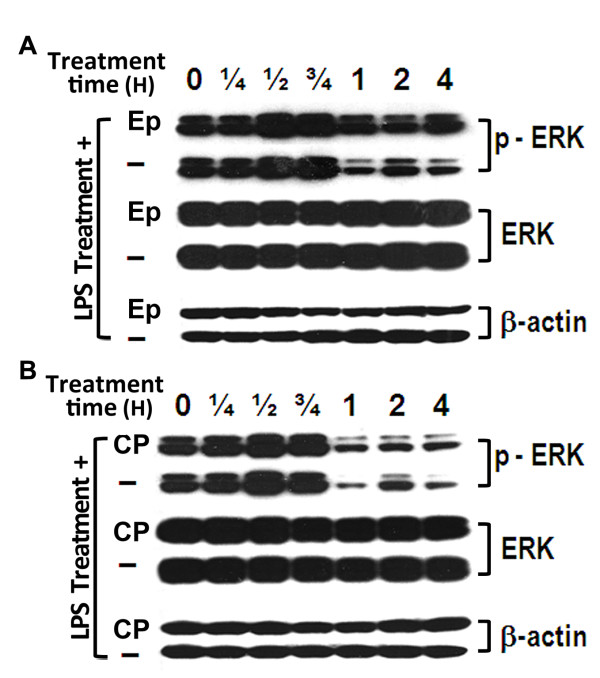
**Delay in inactivation of the ERK pathway during the mid-stage of LPS stimulation**. The phosphorylation levels of ERK1/2 were determined by western blotting analysis after LPS-stimulated THP-1 cells were co-treated with **(A) **BF/S+L/Ep (Ep) or **(B) **cytopiloyne (CP).

Since phosphorylation of ERK1/2 plays a pivotal role during the activation of ERK signaling pathway [5,22], we used western blot analysis to test our hypothesis that ERK1/2 is a key molecular target for the actions of both BF/S+L/Ep and cytopiloyne (Figure 5B). During LPS stimulation in THP-1 cells, the phosphorylation level of ERK1/2 molecules was first induced between 0.5 and 0.75 h post-treatment, it was then suppressed between 1 and 4 h post-treatment (Figure 8A, 8B). When LPS-stimulated THP-1 cells were co-treated with test BF/S+L/Ep (Figure 8A) or cytopiloyne (Figure 8B), a similar trend of activation and suppression of phosphorylation of ERK1/2 was observed, but the exact pattern and the level of dephosphorylation of ERK1/2 in phytocompound-treated cells were substantially different, resulting in a delay in the dephosphorylation time course for ERK1/2 in BF/S+L/Ep or cytopiloyne-treated cells. Densitometer analyses showed a 2-3 fold change in phosphorylated ERK1/2 levels for cytopiloyne and (BF/S+L/Ep) between 1 and 4 h post-treatment.

Cascade network activities conferred by emodin and BF/S+L/Ep (Figure 6B, C) were hypothesized to be mediated by signaling or function via E6-AP, a key member of the E3 ligase enzymes involved in ubiquitination pathways. We therefore also tested the ubiquitination activity in THP-1 cells treated with or without these two test phytocompounds. By using a ubiquitin enrichment assay to determine polyubiquitin levels of total proteins in test cell samples, we were unable in this case to detect a difference between phytocompound-treated and untreated (control) cells (data not shown). This result, however, has not ruled out the possibility that emodin or BF/S+L/Ep can preferentially affect ubiquitination activity in a specific manner via E6-AP activity. Possible specific subset(s) of protein substrates in test cells that may be affected in such a manner by test phytocompounds will need to be evaluated in future systematic studies.

## Discussion

In this study, we characterized the immunomodulatory pharmacogenomics of phytocompounds and herbal extracts (shikonin, emodin, cytopiloyne and BF/S+L/Ep) on gene expression profiles in LPS-induced THP-1 cells, a well established immune cell line, using a transcriptome approach. A number of key microarray results obtained from LPS-only treatment were confirmed in this study by RT-PCR assay and pathway analysis (Figure 2C, 5A). Our experimental results are thus verified in general with overlapping and cross-referencing in data for expression of several key test genes. With this baseline information available, we found that shikonin and emodin repress cytokine, chemotaxis and cell migration genes through interference with the ubiquitin pathway. BF/S+L/Ep and cytopiloyne showed a striking similarity in their patterns of regulation of immune-related gene expression, suggesting the presence of compounds with similar activity to cytopiloyne in the *Echinacea purpurea *preparations. Hence, co-treatment of THP-1 cells with LPS and shikonin, emodin or other phytocompounds was designed here to evaluate the very early response or even a prevention/blockade activity of an inflammatory response, in reaction to LPS, which serves as a common inflammatory stimulator.

Using a structured network knowledge-based approach to analyze genome-wide transcriptional responses, Calvano recently reported that specific functional modules, defined as typical innate immune activities, in human blood leukocytes are highly responsive to inflammatory endotoxin stimulation *in vivo*. Our current *in vitro *investigation into the transcriptional response of a monocyte/macrophage cell system, using a focused DNA microarray with a smaller subset of immune-related genes, has found interesting similarities in the effect of early and medium innate immune gene expressions involved in the inflammatory response. As an example, the expression of proinflammatory cytokines and chemokines (TNF, IL-1 beta, CCL2 and IL-8) reached a peak during the early (0.5 h) and medium (4 h) stages of inflammatory response. Therefore, the findings obtained in this study are complementary to and consistent with the previous *in vivo *studies [23].

It has been shown that quiescent/inactive monocytes or macrophages do not express IL2RA (the alpha chain of IL-2 receptor); however, expression of IL2RA gene has been shown to be inducible after activation of human peripheral blood monocytes [24-26]. The molecular mechanism for IL2RA gene regulation by *M. tuberculosis*, a gram-negative bacterium, has been shown to be mediated via activation of NF-κB in THP-1 cells, our test cells [27]. Therefore, it is possible that IL2RA expression can occur in THP-1 cells. Nonetheless, although we have shown in this study that IL2RA mRNA expression is down-regulated in LPS-stimulated THP-1 cells after treatment with shikonin or emodin, the down-regulation does not necessarily correlate with a decrease in protein levels of IL2RA in test cells treated with phytocompounds, as post-transcriptional and post-translational modifications, including regulation via microRNAs and ubiquitin/proteosome pathways, are well known to affect protein expression of a target mRNA. An example is the effect of shikonin on TNF-α gene expression, as we have previously shown in THP-1 cells [9]. Hence future study is needed to address this possibility.

This relatively small-scale, function-targeted focused DNA microarray set and the specific time course design, when employed to distinguish the effect of different phytocompounds/medicinal herbal extracts on the patterns of kinetic gene expression, is hence a useful strategy for analyzing the specific molecular mechanisms involved in the bioactivities of various candidate herbal medicines. Since the LPS-stimulated THP-1 cell system is one of the most widely-used models for macrophage activation, our findings on the differential effects of various phytocompounds on anti-inflammatory activities may thus have a general application. In this study, we have used THP-1 cells that were not treated with LPS as an internal control. It is important to note here that the response of THP-1 to LPS obtained in the present study is in good agreement with various reports from previous studies [3,15-17,23]. We then compared data obtained in the set from treatment with LPS only with the vehicle control set (i.e., without LPS treatment, data not shown) and further analyzed the gene expression patterns (Figure 2C) and the possible signaling pathways or potential interactions involved (Figure 5A). The study was hence designed as a pharmacogenomics approach for evaluation and classification of various anti-inflammatory natural products, mainly phytochemicals with reputed medicinal bioactivities, into potentially clinically relevant or applicable subgroups.

Shikonin and emodin resulted in significant inhibition (to less than 30% of control) of most of the inflammation responsive genes as early as 0.5 h after treatment (Table 1). Shikonin has previously been shown to exhibit anti-inflammatory activity [28], and here we also observed shikonin suppression of the expression of a number of immune-related genes, including TNF-α, IL-1β and CCL4 genes. This suggests that shikonin can inhibit a group of genes that are associated with macrophage activation at the very early stage of inflammatory response.

Following emodin treatment, approximately 30 genes were significantly down-regulated even after 48 h (data not shown). However, there was less emodin inhibition of some chemotactic and cell migration gene expression, such as CCBP2 and CCL4 compared to shikonin (Table 1). This suggests that emodin might down-regulate inflammatory cytokines rather than immune cell recruitment and cell migration. Recently, emodin has been shown to exert an anti-inflammatory effect by inhibiting NF-κB activation and inflammatory cytokine expression [10,11]. We suggest that both shikonin and emodin may strongly inhibit macrophage activation, but that chemotaxis and the recruitment of T lymphocytes are less affected by emodin. Further experiments are necessary to confirm this possibility.

We analyzed possible signaling pathways and modulators using key node analysis of those genes significantly inhibited (≥3-fold) by shikonin and emodin treatments (Figure 6A, B respectively) in LPS induced THP-1 cells at the 0.5 h time point (Table 1). The pattern of down-regulated gene expression seen with shikonin at 0.5 h suggested an increase in expression of Rad23A, which binds and delivers ubiquitinated proteins to the proteasome, and subsequent inactivation of the p-300, transcriptional co-activator protein. The down-regulated LPS gene expression pattern following emodin suggested that the activity of Ub protein ligase E6-AP may be blocked at 0.5 h. These ubiquitination and de-ubiquitination mechanisms are emerging as important regulators of Toll-like receptor (TLR) signaling. Recent findings on TLR signaling pathways have shown that Ub is a key molecule of the NF-κB inhibitory proteins that can prevent the formation of signaling complexes [22,29,30]. Therefore, interfering with ubiquitination activity may prove to be a useful strategy for developing therapeutics targeting severe inflammatory diseases. We show here that both shikonin and emodin may act as immediate-early inhibitors of inflammation through interfering with ubiquitin pathways, their use as anti-inflammatory remedy may warrant future evaluation, especially since we have shown that shikonin can be very effective *in vivo *in wound-healing activities in skin tissue [31].

Although BF/S+L/Ep did not inhibit the early macrophage-activation stage at 0.5 h, high suppression of gene expression was however observed at 12 h (Figure 6C, Table 3), which continued for up to 48 h (data not shown). TRANSPATH database analysis suggested that the level of ubiquitination of Rad23A regulated by Ub protein ligase may be increased. This indicates that BF/S+L/Ep may not have strong inhibitory activity in the early stage of the immune response and may be more immunomodulatory than immunosuppressive.

On the other hand, although very few immune-related genes were strongly affected by cytopiloyne and BF/S+L/Ep, the gene expression pattern of these two treatments displayed an obvious similarity. The resemblance between BF/S+L/Ep and cytopiloyne treatments was even more evident in analysis of the time profile of the gene expression ratio compared to LPS stimulation, which was characterized by an up-regulation of gene expression after 4 h of stimulation (Figure 3A and 3B, Table 2). Despite the overall similarity, cytopiloyne showed some mechanistic differences contributing to the delayed down-regulation of genes at 2 h, which was not seen in the BF/S+L/Ep treatment.

To study the detailed mechanism responsible for the similar effects of BF/S+L/Ep and cytopiloyne treatment, we compared the expression profiles of those genes that shared common regulation modes between the two treatments. BF/S+L/Ep and cytopiloyne did not show any significant differences in the "up" group, whereas there were significant differences in the "down" group (Figure 4A). The same scenario was observed with the genes displaying the "early no-response followed by up-regulation" mode (Figure 4B). This analysis further supports the idea that both Asteraceae preparations may affect common master regulator(s) to modulate the expression of immune genes, which are up-regulated at 4 h, and alleviate the down-regulation of genes inhibited by LPS stimulation.

We then analyzed these groups of genes using the TRANSPATH database, which identified the ERK1/2 pathway as a common key regulator at no more than 4 hierarchical levels of gene regulation (Figure 5B). We confirmed that the ERK1/2 pathway was a regulator by western blot analysis experiments (Figure 8A, B) that showed a delay in inactivation of ERK1/2 activities in THP-1 cells when they were treated with BF/S+L/Ep or cytopiloyne. ERK1/2 activation has long been recognized as a pivotal regulation in macrophage activation and cytokine expression during inflammatory responses [32,33]. ERK1/2 molecules are phosphorylated on the threonine and tyrosine residues within minutes of TLR-4 stimulation of macrophages and dendritic cells, as shown via treatment with LPS [34,35]. Our data showing retarded dephosphorylation of ERK1/2 between 2 and 4 h may help to explain the up-regulation of several groups of gene expression at 4 h (Table 2) when test cells were treated with BF/S+L/Ep or cytopiloyne. Recent studies have shown that inactivation of MAPK occurs primarily through regulation via dephosphorylation. The mitogen-activated protein kinase phosphatase (MKP) family includes serine-threonine phosphatases (PP2A and PP2C), protein tyrosine phosphatases (PTPN5, PTPN7, and PTPRR) [36,37], and members of the dual-specificity phosphatases (DUSP) family [35]. There is considerable evidence from both animal model and human studies that pharmacological inhibition of ERK activation may help modify inflammatory responses for clinical applications [34]. Since our data suggest that cytopiloyne and BF/S+L/Ep can effectively interfere with the dephosphorylation status of ERK1/2, the DUSPs may thus represent one of the most likely candidates for such activity.

This study and our previous reports [9,13,14,20] have shown that some Asteraceae plant preparations have very desirable pharmacological properties, including low cell toxicity, anti-inflammatory bio-activity, and a high specific index. Therefore, the current finding on the mechanistic explanation of the Asteraceae preparations action on ERK regulation warrants further investigation. Interestingly, cytopiloyne also possesses the unique ability to delay the suppression of genes downstream of the Lck pathway (Figure 7). The LPS-induced NF-κB pathway depends on phosphorylation of IκB-β, and Src tyrosine kinases such as cSrc and Lck, which are key components of the LPS signaling pathway [4,7]. This suggests that cytopiloyne might affect NF-κB activation through interference with Lck.

## Conclusions

We used a functional genomics approach to characterize and compare the mechanisms and kinetics of immune modulation of LPS-stimulated THP-1 cells by a range of anti-inflammatory phytocompounds, including shikonin, emodin, *Echinacea *extract and cytopiloyne. Shikonin and emodin exhibit immediate early inhibitory activities, apparently by interfering with the ubiquitin pathway. Comparative analysis further showed that BF/S+L/Ep and cytopiloyne shared a similar mode of modulation of immune-related gene expression during acute inflammation, and mode-clustering analysis suggested that the ERK1/2 activation pathway was the target of both cytopiloyne and BF/S+L/Ep. These findings may suggest the presence of active compound(s) related to cytopiloyne in *Echinacea purpurea *preparations [20], and offer mechanistic insight for possible development of these phytocompounds as defined therapeutic agents. We also suggest that specific and structurally different phytocompounds/extracts may exert their immune modulatory effects through recruitment of a number of common signaling networks of immune-responsive genes that warrant future systematic investigation.

## Methods

### Cell culture and monocyte preparation

The human myelogenic leukemia cell line THP-1 was purchased from American Type Culture Collection (Rockville, MD). Cell cultures were maintained in RPMI 1640 (Invitrogen, Carlsbad, CA) supplemented with 10% fetal bovine serum (Life Technologies, Rockville, MD), penicillin (100 U/ml) (Life Technologies), and streptomycin (100 μg/ml) (Life Technologies) at 37°C in 5% CO_2 _in a humidified incubator.

### Preparation of phytocompounds

Shikonin was purchased from TCI (Tokyo, Japan), emodin was purchased from Acros Organics (Morris Plains, NJ), and cytopiloyne was isolated as described previously [12] and provided by the Metabolomics Core Laboratory of the Agricultural Biotechnology Research Center, Academic Sinica.

### Cell viability assay

Cell viability was assayed by MTT colorimetric dye reduction method as described previously [14]. Extracts or phytocompounds tested were serially diluted: shikonin (0.1, 0.5, 1 and 10 μM); emodin (1, 10, 50 and 100 μM); cytopiloyne (1, 10, 50 and 100 μM); BF/S+L/Ep (1, 10, 100, and 200 μg/ml). Throughout our experiments, LPS was used at 1 μg/ml in test culture medium for stimulation of THP-1 cells.

### RNA isolation

1 × 10^7 ^THP-1 cells were transferred to a 10 cm Petri dish in 10 ml culture medium. After incubation overnight, test phytocompounds and LPS were added, and cells were then harvested at different time points. THP-1 cells were collected and pelleted in a microcentrifuge at 900 rpm, and the culture medium supernatant removed. Pelleted THP-1 cells were lyzed with Trizol reagent and extracted with chloroform. The upper aqueous phase was collected by centrifugation at 4°C, 14000 rpm for 15 minutes, and RNA was precipitated from solution by the addition of an equal volume of isopropanol. RNA pellets were washed twice with 75% ethanol/DEPC, and dissolved in DEPC-treated water. Concentration and quality of the RNA samples was analyzed by absorbance at 260/280 nm, before they were stored at -80°C.

### RNA electrophoresis

Aliquots of 2 μl RNA sample (5 μg total RNA) were added to 10 μl of a glyoxal reaction mixture in a closed microcentrifuge tube, incubated at 55°C for 1 hour and then chilled on ice for 2 min, when the aqueous droplets condensed on the wall of the microcentrifuge tube were spun down. RNA samples made up in 1× BTPE buffer (10 mM PIPES, 30 mM Bis-Tris, 0.1 mM EDTA, pH 6.5) were loaded onto a 6 cm-long 1% agarose gel, and electrophoresed in 1× BTPE buffer at 100 V for 15 minutes. Gels were photographed without additional staining.

### Primer design and RT-PCR conditions

DNA primers used to investigate the effect of phytocompounds on the expression of TNF-α, IL-1β and IL-8 RNA contained the following sequences: Human TNF-α sense primer: 5'-CAGGCAGTCAGATCATCTTCTCGAAC-3'. Human TNF-α anti-sense primer: 5'-CGTTTGGGAAGGTTGGATGTTCGTCC-3'. Human IL-1β sense primer: 5'-CCCCAGCCCTTTTGTTGA-3'. Human IL-1β anti-sense primer: 5'-TTCTTGCCCCCTTTGAATAAATT-3'. Human IL-8 sense primer: 5'-CTTGGCAGCCTTCCTGATTT-3'. Human IL-8 anti-sense primer: 5'-CTCAGCCCTCTTCAAAAACT-3'. Human GAPDH sense primer: 5'-GAAGGTGAAGGTCGGAGTC-3'. Human GAPDH anti-sense primer: 5'-GAAGATGGTGATGGGATTTC-3'. RT-PCR reactions used the AccessQuick RT-PCR system (Promega U.S., Madison, WI) according to the manufacturer's instructions. Briefly, 1 μg of total RNA from each sample was added to the reaction mixture containing 1× AccessQuick master mix (*Tfl *DNA polymerase, AMV/*Tfl *reaction buffer, 25 mM MgSO_4 _and 10 mM dNTP mixture), 10 μM each of specific sense and anti-sense primers, 5U AMV reverse transcriptase, and nuclease-free water to a final volume of 50 μl. Reactions were incubated at 48°C for 60 min, and PCR amplification was carried out after denaturing at 95°C for 2 min. Images were scanned and densitometer analysis of the captured image was performed with BIO-1 D image analysis software. The signal intensities of test genes in different samples were normalized to the respective mouse GAPDH signal intensity.

### DNA microarray

The focused, immune function-targeted DNA microarray system was constructed using synthesized oligonucleotide probes as described in our previous study [14]. Briefly, 228 immune function-associated genes were selected and grouped into specific cellular immunological functions, such as chemotaxis, antigen processing, maturation and signaling in dendritic cells, apoptosis, and other immune-related activities. A number of non-immune related, functional genes (e.g., metabolism), and housekeeping genes were also included. Oligonucleotide probes were designed and synthesized with a length of approximately 50 nucleotides to represent specifically these genes as defined by the U-GET program as reported by Iyer et al. [38] and our previous studies [14,39]. This gene list is now freely available to the public scientific community upon request (Additional file 1: Table S1). A two color CyDye system was used for determining the ratio of gene expression for test/control set sample after different treatments.

### Reverse transcription and first strand cDNA labeling with amino allyl-dUTP (AA-dUTP)

A total RNA sample (20 μg) was mixed with 3 μl of Oligo dT primer (Amersham Pharmacia Biotech, Buckinghamshire, UK) and heated at 70°C for 5 minutes, then allowed to cool for 10 minutes at room temperature. The reaction mixture (total volume 20 μl), containing 4 μl of 5X first-strand buffer, 2 μl of 0.1 M DTT, 1 μl of a 20X nucleotide mixture, 1 μl AA-dUTP and 1 μl of reverse transcription reaction, was incubated at 42°C for 1.5 h. The reaction was terminated by addition of 2 μl of 2.5 M NaOH and followed by incubation at 37°C for 15 min. The reaction mixture was neutralized with 10 μl of 2 M HEPES and the synthesized cDNA product was cleaned up with a Microcon purification kit (Millipore, YM30). The cDNA pellet was then speed-vacuum dried and resuspended in 15 μl water.

### Labeling of amino allyl-modified cDNA with CyDye

An aliquot of CyDye (Cy3 or Cy5, GE Healthcare) was resuspended in 15 μl fresh 0.1 M NaHCO_3 _pH 9.0, immediately prior to use in a labeling reaction. One aliquot of resuspended CyDye was then added to one tube of AA-dUTP-modified cDNA (see above) using Cy3 for control samples, and Cy5 for treated samples. Tubes were mixed by stirring, and incubated at room temperature in the dark for 1 hour, after which 15 μl 4-hydroxylamine was added to each coupling reaction, mixed well and incubated at room temperature in the dark, for a further 15 minutes. CyDye-labeled cDNA was then purified using a DNA purification kit (Qiagen). The allyl-modified cDNA pellet was then speed-vacuum dried and resuspended in 5 μl water.

### Hybridization

A probe was prepared in fresh hybridization solution consisting of 30% formamide, 5X SSC, 0.1% SDS, and 0.1 mg/ml of a nucleic acid blocker, human Cot1 DNA. The probe was incubated in solution at 95°C for 5 minutes, then lightly centrifuged for 2 minutes to collect condensation, and cooled to room temperature for 20 minutes, and placed in a hybridization chamber (Corning, cat. No.2551). The probe was then pipetted onto the printed surface of the slide. A coverslip was carefully placed on top of the array to avoid bubble formation during hybridization. The chamber was placed in a 42°C water bath for 16 hours.

### Post-hybridization washing

The array was washed in 2× SSC, 0.1% SDS at 42°C for 5 minutes, and then in a second buffer containing 0.1× SSC, 0.1% SDS at room temperature for 5 minutes, and the process was repeated once. The array was then washed 4 times in 0.1× SSC buffer at room temperature for 1 minute. The array was then dried by centrifugation (800 rpm, 10 min), and the signal emitted from each spot was analyzed with digital imaging software (Genepix 4.0, Axon Instruments, MDS Analytical, Toronto Canada).

### Western blot analysis

Total proteins were extracted from test THP-1 cells with ice-cold lysis buffer (150 mM NaCl, 0.5% Triton X-100, 50 mM Tris-HCl (pH 7.4), 20 mM EGTA, 1 mM dithiothreitol (DTT), and protease inhibitor cocktail) and centrifuged at 12,000 × *g *for 20 min. Protein samples were subjected to western blotting as described previously.^9 ^Briefly, test proteins were assayed after overnight incubation at 4°C with 1:1000 dilution of polyclonal p44/p42 MAPK (ERK1/2) or phosphor-specific ERK1/2 antibodies (Cell Signaling). Equal protein loading was assessed using mouse α-actin (Sigma Chemical Co., St. Louis, MO). The proteins were visualized with an enhanced chemiluminescence (ECL) detection kit (Amersham).

### Data and signaling pathways analysis

The focused array system that we used in this study was adapted from the system reported by Iyer et al. [38] and Wang et al. [14]. We employed Cy3 and Cy5 fluorescent dyes to label the RNA samples obtained from the control and treatment groups, respectively. The Cy3 (control) and Cy5 (test) labeled RNA samples were then mixed and subjected to hybrdization with oligo-nucleotide probes on chips. Five different housekeeping genes, alpha-Tubulin (NM_006082), beta-2-microglobulin (NM_004048), beta-actin (NM_001101), GAPDH (NM_002046), Transferrin R (NM_003234), have been built into the design of our "array genes". These 5 housekeeping genes were hence employed as the "internal controls" of our gene chip assay. Within each array chip, four replicates for each gene were used.

The scanning output generated from the focused arrays was fed into GenePix to extract numerical expression readings from each spot. The relative expression level of each gene was represented by the median of ratio (Cy5/Cy3) averaged from the four replicates of a gene on the same array. As we previously described [14], our microarray data were analyzed using the Spotfire software, which includes established algorithms that determine whether a gene is present or absent and whether the expression level of a gene in specific experimental test samples is significantly increased or decreased (≥ 3-fold change) relative to a control sample, and for clustering distinct groups of gene expression profiles (hierarchical clustering and K-means). The signals obtained from different chips (test sets) were normalized by the relative expression level (in ratio) to the β-actin (B2M) gene. Only those genes that showed at least a 3-fold change in expression level after phytocompound or extract treatment were listed in our study and then further classified into different gene cluster groups and used for analyses of signaling networks [40]. Other measures for data processing, information search and analyses, such as the use of KEGG (Kyoto Encyclopedia of Genes and Genomics; http://www.genome.jp/kegg/ and TRANSPATH, have been briefly described previously [14,20]. The TRANSPATH database offers good details on molecules or networks of the signaling flow from the cell membrane into the nucleus, focusing on mammals such as humans, mice, and rats [21].

## Authors' contributions

SCC designed the experiments, carried out data analyses and wrote the drafted manuscript. SWT served as a key experimenter and contributed the draft manuscript. PIH and YAC as a team performed bioinformatics and signaling pathway analyses on DNA microarray data. VS provided important input into 'Discussion" and made useful revisions and reorganization of the manuscript. NSY is the principal investigator and laboratory head of the study, and is the principal author of the manuscript. All authors read and approved the final manuscript.

## Supplementary Material

Additional file 1 **228 gene list with ontological description**. The list was represented gene IDs, symbols and names of 228 genes with ontological description in our DNA microarray system. Click here for file

## References

[B1] Locksley RM Killeen N Lenardo MJ The TNF and TNF receptor superfamilies: integrating mammalian biology Cell 2001 11 487 501 10.1016/S0092-8674(01)00237-9 11239407

[B2] Janeway CA Jr Medzhitov R Innate immune recognition Annu Rev Immunol 2002 11 197 216 10.1146/annurev.immunol.20.083001.084359 11861602

[B3] Sharif O Bolshakov VN Raines S Newham P Perkins ND Transcriptional profiling of the LPS induced NF-kappaB response in macrophages BMC Immuno 2007 11 1 10.1186/1471-2172-8-1 PMC1781469 17222336

[B4] Harkonen PL Vaananen HK Monocyte-macrophage system as a target for estrogen and selective estrogen receptor modulators Ann N Y Acad Sci 2006 11 218 227 10.1196/annals.1386.045 17261769

[B5] Nilsson R Bajic VB Suzuki H di Bernardo D Björkegren J Katayama S Reid JF Sweet MJ Gariboldi M Carninci P Hayashizaki Y Hume DA Tegner J Ravasi T Transcriptional network dynamics in macrophage activation Genomics 2006 11 133 42 10.1016/j.ygeno.2006.03.022 16698233

[B6] Staniforth V Wang SY Shyur LF Yang NS Shikonins, phytocompounds from *Lithospermum erythrorhizon*, inhibit the transcriptional activation of human tumor necrosis factor alpha promoter in vivo J Biol Chem 2004 11 5877 5885 10.1074/jbc.M309185200 14645256

[B7] Song E Antus B Yao Y Lutz J Heemann U Sequential activation patterns of macrophages in chronic allograft nephropathy Graft 2002 11 141 144 10.1177/1522162802005003005

[B8] Russell SW Doe WF McIntosh AT Functional characterization of a stable, noncytolytic stage of macrophage activation in tumors J Exp Med 1977 11 1511 1520 10.1084/jem.146.6.1511 925611 PMC2181899

[B9] Chiu SC Yang NS Inhibition of tumor necrosis factor-alpha through selective blockade of pre-mRNA splicing by shikonin Mol Pharmaco 2007 11 1506 1512 10.1124/mol.106.032821 17360831

[B10] Chen RF Shen YC Huang HS Liao JF Ho LK Chou YC Wang WY Chen CF Evaluation of the anti-inflammatory and cytotoxic effects of anthraquinones and anthracene derivatives in human leucocytes J Pharm Pharmacol 2004 11 915 919 10.1211/0022357023781 15233871

[B11] Li HL Chen HL Li H Zhang KL Chen XY Wang XW Kong QY Liu J Regulatory effects of emodin on NF-kappaB activation and inflammatory cytokine expression in RAW 264.7 macrophages Int J Mol Med 2005 11 41 47 15942676

[B12] Chiang YM Chang CL Chang SL Yang WC Shyur LF Cytopiloyne, a novel polyacetylenic glucoside from *Bidens pilosa*, functions as a T helper cell modulator J Ethnopharmacol 2007 11 532 8 10.1016/j.jep.2006.10.007 17101254

[B13] Chang CL Chang SL Lee YM Chiang YM Chuang DY Kuo HK Yang WC Cytopiloyne, a polyacetylenic glucoside, prevents Type 1 diabetes in nonobese diabetic mice J Immunol 2007 11 6984 6993 17513748 10.4049/jimmunol.178.11.6984

[B14] Wang CY Chiao MT Yen PJ Huang WC Hou CC Chien SC Yeh KC Yang WC Shyur LF Yang NS Modulatory effects of *Echinacea purpurea *extracts on human dendritic cells: a cell- and gene-based study Genomics 2006 11 801 808 10.1016/j.ygeno.2006.08.011 17011161

[B15] Harrison LM van den Hoogen C van Haaften WC Tesh VL Chemokine expression in the monocytic cell line THP-1 in response to purified shiga toxin 1 and/or lipopolysaccharides Infect Immun 2005 11 403 412 10.1128/IAI.73.1.403-412.2005 15618178 PMC538957

[B16] Mikita T Porter G Lawn RM Shiffman D Oxidized low density lipoprotein exposure alters the transcriptional response of macrophages to inflammatory stimulus J Biol Chem 2001 11 45729 45739 10.1074/jbc.M106114200 11577090

[B17] Yang S Tamai R Akashi S Takeuchi O Akira S Sugawara S Takada H Synergistic effect of muramyldipeptide with lipopolysaccharide or lipoteichoic acid to induce inflammatory cytokines in human monocytic cells in culture Infect Immun 2001 11 2045 2053 10.1128/IAI.69.4.2045-2053.2001 11254557 PMC98129

[B18] Beutler B Rietschel ET Innate immune sensing and its roots: the story of endotoxin Nat Rev Immunol 2003 11 169 176 10.1038/nri1004 12563300

[B19] Le Naour F Hohenkirk L Grolleau A Misek DE Lescure P Geiger JD Hanash S Beretta L Profiling changes in gene expression during differentiation and maturation of monocyte-derived dendritic cells using both oligonucleotide microarrays and proteomics J Biol Chem 2001 11 17920 17931 10.1074/jbc.M100156200 11279020

[B20] Wang CY Staniforth V Chiao MT Hou CC Wu HM Yeh KC Chen CH Hwang PI Wen TN Shyur LF Yang NS Genomics and proteomics of immune modulatory effects of a butanol fraction of *Echinacea purpurea *in human dendritic cells BMC Genomics 2008 11 479 10.1186/1471-2164-9-479 18847511 PMC2571112

[B21] Krull M Pistor S Voss N Kel A Reuter I Kronenberg D Michael H Schwarzer K Potapov A Choi C Kel-Margoulis O Wingender E TRANSPATH: an information resource for storing and visualizing signaling pathways and their pathological aberrations Nucleic Acids Res 2006 34 Database D546 D551 10.1093/nar/gkj107 16381929 PMC1347469

[B22] Ea CK Deng L Xia ZP Pineda G Chen ZJ Activation of IKK by TNF-α requires site-specific ubiquitination of RIP1 and polyubiquitin binding by NEMO Mol Cell 2006 11 245 257 10.1016/j.molcel.2006.03.026 16603398

[B23] Calvano SE Xiao W Richards DR Felciano RM Baker HV Cho RJ Chen RO Brownstein BH Cobb JP Tschoeke SK Miller-Graziano C Moldawer LL Mindrinos MN Davis RW Tompkins RG Lowry SF Inflammation and Host Response to Injury Large Scale Collab Res Program A network-based analysis of systemic inflammation in humans Nature 2005 11 1032 1037 10.1038/nature03985 16136080

[B24] Holter W Goldman CK Casabo L Nelson DL Greene WC Waldmann TA Expression of functional IL 2 receptors by lipopolysaccharide and interferon-g stimulated human monocytes J Immunol 1987 11 2917 2922 3106493

[B25] Holter W Grunow R Stockinger H Knapp W Recombinant interferon-g induces interleukin 2 receptors on human peripheral blood monocytes J Immunol 1986 11 2171 2175 3081639

[B26] Wahl SM McCartney-Francis N Hunt DA Smith PD Wahl LM Katona IM Monocyte interleukin 2 receptor gene expression and interleukin 2 augmentation of microbicidal activity J Immunol 1987 11 1342 1347 3039002

[B27] Tchou-Wong KM Tanabe O Chi C Yie TA Rom WN Activation of NF-kappaB in *Mycobacterium tuberculosis*- induced interleukin-2 receptor expression in mononuclear phagocytes Am J Respir Crit Care Med 1999 11 1323 1329 10194184 10.1164/ajrccm.159.4.9710105

[B28] Chen X Yang L Oppenheim JJ Howard MZ Cellular pharmacology studies of shikonin derivatives Phytother Res 2002 11 199 209 10.1002/ptr.1100 12164262

[B29] Hayden MS Ghosh S Signaling to NF-κB Genes Dev 2004 11 2195 2224 10.1101/gad.1228704 15371334

[B30] Wullaert A Heyninck K Janssens S Beyaert R Ubiquitin: tool and target for intracellular NF-kappaB inhibitors Trends Immunol 2006 11 533 540 10.1016/j.it.2006.09.003 16982211

[B31] Wang JH Lin KF Benson SA Sun SJ Cheng WM Wang SY Shyur LF Yang NS Tissue array transgene expression system for the evaluation of effect of medicinal herbs on wound-healing J Gen Mol Biol 2003 11 133 144

[B32] Dumitru CD Ceci JD Tsatsanis C Kontoyiannis D Stamatakis K Lin JH Patriotis C Jenkins NA Copeland NG Kollias G Tsichlis PN TNF-alpha induction by LPS is regulated posttranscriptionally via a Tpl2/ERK-dependent pathway Cell 2000 11 1071 1083 10.1016/S0092-8674(00)00210-5 11163183

[B33] Mahtani KR Brook M Dean JL Sully G Saklatvalsa J Clark AR Mitogen-activated protein kinase p38 controls the expression and posttranslational modification of tristetraprolin, a regulator of tumor necrosis factor mRNA stability Mol Cell Biol 2001 11 6461 6469 10.1128/MCB.21.9.6461-6469.2001 11533235 PMC99793

[B34] Jeffrey KL Camps M Rommel C Mackay CR Targeting dual-specificity phosphatases: manipulating MAP kinase signalling and immune responses Nat Rev Drug Discov 2007 11 391 403 10.1038/nrd2289 17473844

[B35] Lang R Hammer M Mages J DUSP meet immunology: dual specificity MAPK phosphatases in control of the inflammatory response J Immunol 2006 11 7497 7504 17114416 10.4049/jimmunol.177.11.7497

[B36] Berenson LS Yang J Sleckman BP Murphy TL Murphy KM Selective requirement of p38α MAPK in cytokine-dependent, but not antigen receptor-dependent, TH1 responses J Immunol 2006 11 4616 4621 16585552 10.4049/jimmunol.176.8.4616

[B37] Rincon M Flavell RA Davis RA The JNK and p38 MAP kinase signaling pathways in T cell-mediated immune responses Free Radic Biol Med 2000 11 1328 1337 10.1016/S0891-5849(00)00219-7 10924852

[B38] Iyer VR Eisen MB Ross DT Schuler G Moore T Lee JC Trent JM Staudt LM Hudson J Jr Boguski MS Lashkari D Shalon D Botstein D Brown PO The transcriptional program in the response of human fibroblasts to serum Science 1999 11 83 87 10.1126/science.283.5398.83 9872747

[B39] Yang NS Shyur LF Chen CH Wang SY Tzeng CM Medicinal herb extract and a single-compound drug confer similar complex pharmacogenomic activities in mcf-7 cells J Biomed Sci 2004 11 418 422 10.1007/BF02254447 15067226

[B40] Jaeger J Spang R Selecting normalization genes for small diagnostic microarrays BMC Bioinformatics 2006 11 388 10.1186/1471-2105-7-388 16925821 PMC1560169

