# Supplementary material for: Differential functional genomic effects of anti-inflammatory phytocompounds on immune signaling
Source: BMC Genomics. 2010 Sep 24;11:513. doi: 10.1186/1471-2164-11-513 (PMC2997007; doi:10.1186/1471-2164-11-513)
Supplement: Additional file 1 — 228 gene list with ontological description. The list was represented gene IDs, symbols and names of 228 genes with ontological description in our DNA microarray system. [file 1471-2164-11-513-S1.DOC]

positive regulation of cell proliferation, cell

surface receptor linked signal transduction

Interleukin 12 receptor, beta 2

IL12RB2

3595

signal transduction, leukocyte adhesion,

regulation of transcription, DNA

-

dependent,

response to virus, apoptosis, anti

-

apoptosis

Tumor necrosis factor (TNF

superfamily

,

member 2)

TNF

7124

negative regulation of cell proliferation,

chemotaxis

,

neutrophil

activation, intracellular

signaling cascade, angiogenesis, cell motility,

G

-

protein coupled receptor protein signaling

pathway, calcium

-

mediated signaling

Interleukin 8

IL8

3576

inflammatory response

Interleukin 5 (colony

-

stimulating factor,

eosinophil

)

IL5

3567

inflammatory response, cell motility,

antimicrobial

humoral

response (

sensu

Vertebrata)

interleukin 13

IL13

3596

cell adhesion

Integrin

, beta 7

ITGB7

3695

Guanylate

binding protein 1, interferon

-

inducible, 67kDa

GBP1

2633

signal transduction, cell

-

cell signaling,

induction of apoptosis

tumor necrosis factor (

ligand

)

superfamily

,

member 10

TNFSF10

8743

transcription from RNA polymerase II promoter

CCAAT/enhancer binding protein

(C/EBP), delta

CEBPD

1052

protein amino acid

glycosylation

Fucosyltransferase

7 (alpha (1,3)

fucosyltransferase

)

FUT7

2529

Lymphocyte

-

activation gene 3

LAG3

3902

signal transduction

Mitogen

-

activated protein

kinase

7

MAPK7

5598

Chromosome 1 open reading frame 61

C1orf61

10485

Pleckstrin

homology, Sec7 and coiled

-

coil

domains, binding protein

PSCDBP

9595

inflammatory response, 'regulation of

transcription from RNA polymerase II promoter

Nuclear factor of activated T

-

cells,

cytoplasmic

,

calcineurin

-

dependent 3

NFATC3

4775

transcription from RNA polymerase II promoter

nuclear factor of activated T

-

cells,

cytoplasmic

,

calcineurin

-

dependent 1

NFATC1

4772

regulation of transcription, DNA

-

dependent

Nuclear factor of activated T

-

cells,

cytoplasmic

,

calcineurin

-

dependent 2

NFATC2

4773

cellular defense response

IL2

-

inducible T

-

cell

kinase

ITK

3702

nuclear factor of activated T

-

cells,

cytoplasmic

,

calcineurin

-

dependent 2

interacting protein

NFATC2IP

84901

regulation of transcription from RNA

polymerase II promoter

Jun B proto

-

oncogene

JUNB

3726

transcription from RNA polymerase II promoter

v

-

maf

musculoaponeurotic

fibrosarcoma

oncogene

homolog (avian)

MAF

4094

defense response, transcription from RNA

polymerase II promoter, morphogenesis

GATA binding protein 3

GATA3

2625

regulation of transcription, DNA

-

dependent,

development

T

-

box 21

TBX21

30009

transcription from RNA polymerase II promoter

ets

variant gene 1

ETV1

2115

signal

transduction,development,protein

amino

acid

phosphorylation,cell

proliferation

v

-

ets

erythroblastosis

virus E26

oncogene

like (avian)

ERG

2078

**gene ontology description**

**Name**

**Gene**

**Symbol**

**Gene**

**ID**

**Table S1: 228 gene list with ontological description**

positive regulation of cell proliferation, cell

surface receptor linked signal transduction

Interleukin 12 receptor, beta 2

IL12RB2

3595

signal transduction, leukocyte adhesion,

regulation of transcription, DNA

-

dependent,

response to virus, apoptosis, anti

-

apoptosis

Tumor necrosis factor (TNF

superfamily

,

member 2)

TNF

7124

negative regulation of cell proliferation,

chemotaxis

,

neutrophil

activation, intracellular

signaling cascade, angiogenesis, cell motility,

G

-

protein coupled receptor protein signaling

pathway, calcium

-

mediated signaling

Interleukin 8

IL8

3576

inflammatory response

Interleukin 5 (colony

-

stimulating factor,

eosinophil

)

IL5

3567

inflammatory response, cell motility,

antimicrobial

humoral

response (

sensu

Vertebrata)

interleukin 13

IL13

3596

cell adhesion

Integrin

, beta 7

ITGB7

3695

Guanylate

binding protein 1, interferon

-

inducible, 67kDa

GBP1

2633

signal transduction, cell

-

cell signaling,

induction of apoptosis

tumor necrosis factor (

ligand

)

superfamily

,

member 10

TNFSF10

8743

transcription from RNA polymerase II promoter

CCAAT/enhancer binding protein

(C/EBP), delta

CEBPD

1052

protein amino acid

glycosylation

Fucosyltransferase

7 (alpha (1,3)

fucosyltransferase

)

FUT7

2529

Lymphocyte

-

activation gene 3

LAG3

3902

signal transduction

Mitogen

-

activated protein

kinase

7

MAPK7

5598

Chromosome 1 open reading frame 61

C1orf61

10485

Pleckstrin

homology, Sec7 and coiled

-

coil

domains, binding protein

PSCDBP

9595

inflammatory response, 'regulation of

transcription from RNA polymerase II promoter

Nuclear factor of activated T

-

cells,

cytoplasmic

,

calcineurin

-

dependent 3

NFATC3

4775

transcription from RNA polymerase II promoter

nuclear factor of activated T

-

cells,

cytoplasmic

,

calcineurin

-

dependent 1

NFATC1

4772

regulation of transcription, DNA

-

dependent

Nuclear factor of activated T

-

cells,

cytoplasmic

,

calcineurin

-

dependent 2

NFATC2

4773

cellular defense response

IL2

-

inducible T

-

cell

kinase

ITK

3702

nuclear factor of activated T

-

cells,

cytoplasmic

,

calcineurin

-

dependent 2

interacting protein

NFATC2IP

84901

regulation of transcription from RNA

polymerase II promoter

Jun B proto

-

oncogene

JUNB

3726

transcription from RNA polymerase II promoter

v

-

maf

musculoaponeurotic

fibrosarcoma

oncogene

homolog (avian)

MAF

4094

defense response, transcription from RNA

polymerase II promoter, morphogenesis

GATA binding protein 3

GATA3

2625

regulation of transcription, DNA

-

dependent,

development

T

-

box 21

TBX21

30009

transcription from RNA polymerase II promoter

ets

variant gene 1

ETV1

2115

signal

transduction,development,protein

amino

acid

phosphorylation,cell

proliferation

v

-

ets

erythroblastosis

virus E26

oncogene

like (avian)

ERG

2078

**gene ontology description**

**Name**

**Gene**

**Symbol**

**Gene**

**ID**
